# Neurotensin receptor type 2 protects B-cell chronic lymphocytic leukemia cells from apoptosis

**DOI:** 10.1038/onc.2017.365

**Published:** 2017-10-23

**Authors:** A Abbaci, H Talbot, S Saada, N Gachard, J Abraham, A Jaccard, D Bordessoule, A L Fauchais, T Naves, M O Jauberteau

**Affiliations:** 1 Limoges University, Equipe Accueil 3842, Cellular Homeostasis and Diseases, Faculty of Medicine, Limoges Cedex, France; 2 Hematology Laboratory, Dupuytren Hospital University Center of Limoges, Limoges Cedex, France; 3 CNRS-UMR 7276, Limoges University, Limoges Cedex, France; 4 Department of Hematology, Dupuytren Hospital University Center of Limoges, Limoges Cedex, France; 5 Department of Internal Medicine, Dupuytren Hospital University Center of Limoges, Limoges Cedex, France; 6 Department of Immunology, Dupuytren Hospital University Center of Limoges, Limoges Cedex, France

## Abstract

B-cell chronic lymphocytic leukemia (B-CLL) cells are resistant to apoptosis, and consequently accumulate to the detriment of normal B cells and patient immunity. Because current therapies fail to eradicate these apoptosis-resistant cells, it is essential to identify alternative survival pathways as novel targets for anticancer therapies. Overexpression of cell-surface G protein-coupled receptors drives cell transformation, and thus plays a critical role in malignancies. In this study, we identified neurotensin receptor 2 (NTSR2) as an essential driver of apoptosis resistance in B-CLL. NTSR2 was highly expressed in B-CLL cells, whereas expression of its natural ligand, neurotensin (NTS), was minimal in both B-CLL cells and patient plasma. Surprisingly, NTSR2 remained in a constitutively active phosphorylated state, caused not by a mutation-induced gain-of-function but rather by an interaction with the oncogenic tyrosine kinase receptor TrkB. Functional and biochemical characterization revealed that the NTSR2–TrkB interaction acts as a conditional oncogenic driver requiring the TrkB ligand brain-derived neurotrophic factor (BDNF), which unlike NTS is highly expressed in B-CLL cells. Together, NTSR2, TrkB and BDNF induce autocrine and/or paracrine survival pathways that are independent of mutation status and indolent or progressive disease course. The NTSR2–TrkB interaction activates survival signaling pathways, including the Src and AKT kinase pathways, as well as expression of the anti-apoptotic proteins Bcl-2 and Bcl-xL. When NTSR2 was downregulated, TrkB failed to protect B-CLL cells from a drastic decrease in viability via typical apoptotic cell death, reflected by DNA fragmentation and Annexin V presentation. Together, our findings demonstrate that the NTSR2–TrkB interaction plays a crucial role in B-CLL cell survival, suggesting that inhibition of NTSR2 represents a promising targeted strategy for treating B-CLL malignancy.

## Introduction

Resistance to cell death is often associated with replicative immortality and chemotherapy evasion, two central hallmarks of cancer. This feature is particularly prominent in B-cell chronic lymphocytic leukemia (B-CLL),^1^ in which it leads to the accumulation of malignant mature B lymphocytes.^2^ Several factors have been implicated in CLL development, including mutations in the immunoglobulin heavy-chain variable region gene (*IGHV*) and genomic alterations (deletion, translocation, trisomy and nonsynonymous mutations) used as prognostic markers for indolent or aggressive disease course.^3^ Despite these molecular and genetic findings, however, no curative molecules that can definitively eradicate apoptosis-resistant B-CLL are yet available. Hence, identification of as-yet-unknown mechanisms involved in B-CLL cell survival pathways could provide new targets for anticancer therapies.

Cell-surface receptors, especially G protein-coupled receptors (GPCRs), have emerged as crucial players in malignancies. Several studies show that GPCRs participate actively in signal transduction involved in control of homeostatic processes, including the balance between cell death and proliferation.^4^ In lymphocytes, GPCRs coupled to heterotrimeric G proteins, notably G_i_αs, regulate multiple immune functions and engage in cross-talk with other signaling pathways, including those mediated by tyrosine kinase receptors (TKRs)^5, 6^ such as the Src family of protein kinases (SRC) and the phosphoinositide-3-kinase/protein kinase B (for example, PI3K/AKT).^7^ Thus, mitogen-activated protein kinases (MAPKs), for example, ERK and JNK and the expression of anti-apoptotic proteins belonging to the Bcl-2 family (for example, Bcl-2, Bcl-xL) remain constitutively activated.^8^ These observations suggest that malignant cells can hijack the physiological function of GPCRs for their survival.^9^ Accordingly, GPCR activation might be associated with B-CLL pathogenesis.

To more precisely delineate the potential function of GPCRs in apoptosis evasion by B-CLL cells, we investigated the role of the neurotensin receptors (NTSRs). Two of these proteins, NTSR1 and NTSR2, are GPCRs, whereas NTSR3/sortilin, which has a single transmembrane domain, plays a major role as a sorting receptor.^10^ NTSR1 drives homeostatic processes in the nervous and gastrointestinal systems, but is also actively involved in overexpression of anti-apoptotic proteins and chemotherapy resistance in cancer.^11, 12^

Because NTSR1 is often dysregulated in solid cancers^13^ and previous work by our group showed that NTSRs are differentially expressed in normal and malignant B lymphocytes,^14^ we were interested in studying the potential roles of this GPCR subfamily in B-CLL. In this study, we found that NTSR2, but not NTSR1, was aberrantly overexpressed in B-CLL. Both in NTSR2-overexpressing B-cell models and in cells derived from B-CLL patients, inhibition of NTSR2 by mRNA silencing sensitized cells to apoptosis, pursuant to a decrease in expression of anti-apoptotic proteins. We found that NTSR2 in B-CLL cells is in a constitutively active phosphorylated state, which was reversible by a NTSR2-specific inhibitor. NTSR2 activation is independent of its natural ligand, neurotensin (NTS), and is instead the consequence of its interaction with the TKR tropomyosin-related kinase receptor B (TrkB) and the recruitment of G_i_α proteins. Thus, this complex acts as a conditional oncogene dependent on the TrkB ligand, BDNF, which is highly expressed in B-CLL. We present novel data demonstrating that the NTSR2–TrKB interaction and the sustained activation of the signaling pathways under the control of these two actors constitute an essential driving force for apoptosis evasion in B-CLL.

## Results

### NTSR2 is overexpressed in B-CLL

**Figure 1:**
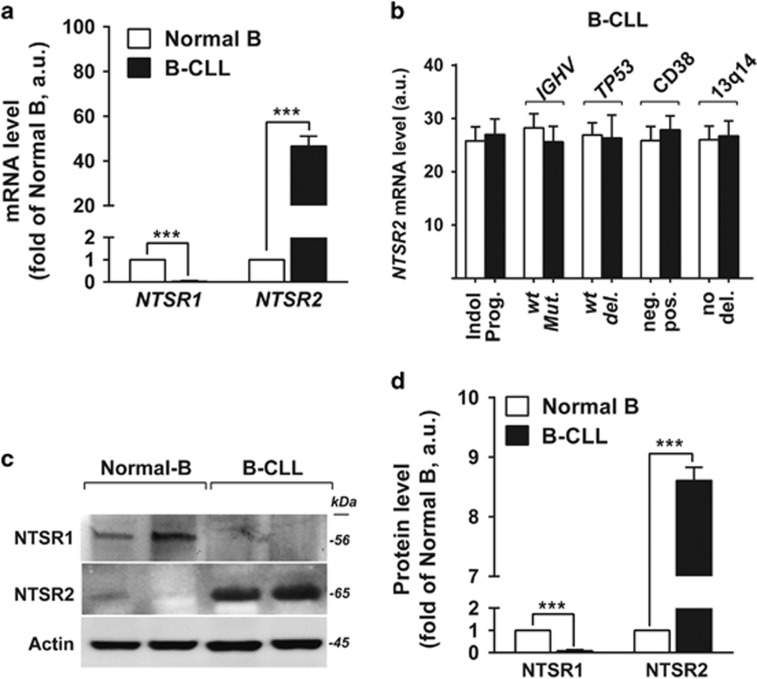
NTSR2, but not NTSR1, is overexpressed in B-CLL. (**a**) Quantitative analyses of *NTSR1* and *NTSR2* mRNA levels in normal B (*n*=15) and B-CLL (*n*=30) lymphocytes, normalized against *HPRT*. Data are expressed as mean fold change in expression (±s.e.m.) vs normal B cells. (**b**) Quantitative analyses of *NTSR2* mRNA level in indolent (Indol.) vs progressive (prog.) patients, wild-type (*wt*) vs mutant (*Mut.*) *IGHV*, wild-type (*wt*) vs mutant (deleted, *del.*) p53, CD38 negative (neg.) vs positive (pos.), and absence (no) vs presence of 13q14 deletion (del.); values are given in arbitrary units (a.u.). (**c**) Representative analysis of NTSR2 expression from B-lymphocyte lysates from two normal donors (D1, D2) and two B-CLL patients (P1, P2). (**d**) NTSR2 expression, normalized against actin, in normal B (*n*=6) and B-CLL (*n*=6) lymphocytes (means±s.e.m. of three independent experiments). Significant *P*-values are indicated in the graphs ***P<0.001.

Based on our previous observation that NTSRs are differentially expressed in malignant human B lymphocytes and drive resistance to Fas ligand-mediated cell death, we speculated that NTSRs contribute to apoptosis resistance in B-CLL pathogenesis. We first compared expression of *NTSR* genes in B lymphocytes between healthy donors (HDs) (Normal B, *n*=15) and B-CLL patients (B-CLL, *n*=30 patients). *NTSR1* was undetectable in B-CLL cells, whereas *NTSR2* was strongly expressed, at levels 30-fold higher (*P*<0.001) than in normal B lymphocytes (Figure 1a). The profile of *NTSR2* expression, which was similar between indolent and progressive B-CLL, was independent of B-CLL markers such as *TP53* deletion, *IGHV* mutation, 13q14 deletion and CD38 expression, indicating that *NTSR2* does not represent a discriminant marker for B-CLL stage (Figure 1b). Analysis of NTSR expression at the protein level confirmed the mRNA results: NTSR2 was overexpressed in B-CLL, whereas NTSR1 was undetectable (Figures 1c and d).

The abnormal expression of NTSR2, independent of B-CLL stage and progression, led us to investigate the roles of the primary mechanisms responsible for GPCR overexpression, that is, gene mutations and chromosomal abnormalities.^9^ To this end, we sequenced *NTSR2* and analyzed the karyotypes of 10 B-CLL patients. None of the B-CLL cases harbored mutations in the *NTSR2* gene, except for two patients with a silent polymorphism (*rs114415067, pH55H*) (data not shown). Moreover, *NTSR2* mutations are present in only 0.3% of the 19 141 samples in COSMIC v62, indicating that *NTSR2* mutations are rare in all combined pathologies.^15^

Together, the significant increase in *NTSR2* mRNA and protein levels suggested that NTSR2 plays a role in the pathogenesis of B-CLL, regardless of the biological and clinical features of this disease.

### NTSR2 depletion sensitizes B-CLL lymphocytes to apoptosis

**Figure 2:**
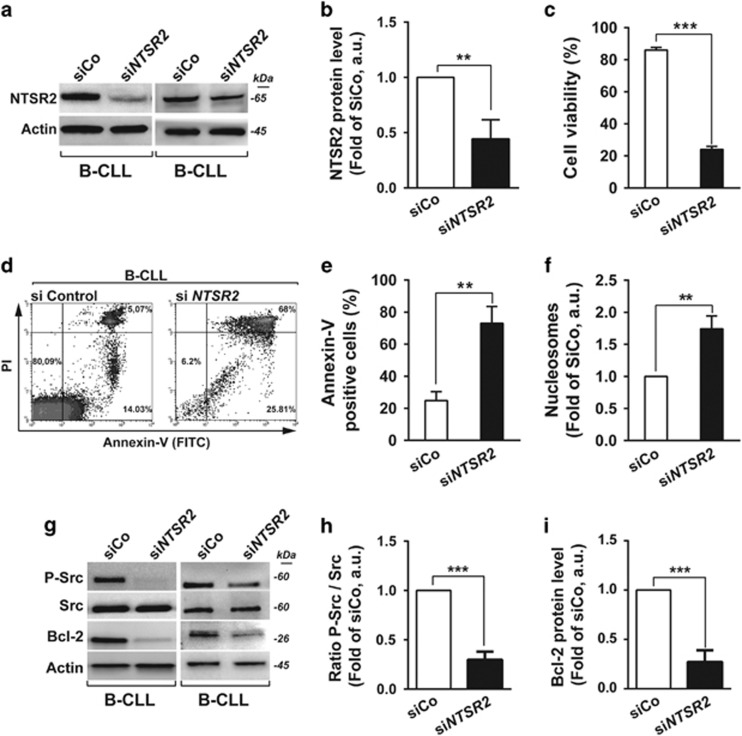
NTSR2 silencing decreases B-CLL viability and induces apoptosis. (**a**) Representative western blot of B-CLL lymphocytes transfected with either a non-relevant siRNA (siRNA control, siCo) or a pool of four different siRNAs directed against NTSR2 (siNTSR2). (**b**) NTSR2 protein level, expressed as fold change vs control (siCo), normalized against actin, in three different B-CLL patients. (**c**) B-CLL cell viability (*n*=4), expressed as a percentage, 72 h after NTSR2 depletion (siCo vs SiNTSR2). (**d**) Representative fluorescence-activated cell sorting analysis of apoptosis induction, assessed by Annexin V-fluorescein Isothiocyanate/PI dual staining of B-CLL cells (*n*=4) depleted or not for NTSR2. (**e**) Percentage of apoptotic cells (Annexin V-positive cells) after *NTSR2* silencing in B-CLL vs siCo. (**f**) Apoptotic ratio in B-CLL cells (*n*=5) 72 h after NTSR2 depletion, assessed by cell death ELISA, expressed as fold change vs control (siCo). (**g**) Representative western blot analysis of p-Src and Bcl-2 expression from B-CLL cell lysates, depleted of *NTSR2* or not for 72 h. (**h**, **i**) Histogram bars represent the fold change in phosphorylation level of Src or Bcl-2 expression in si*NTSR2* cells, normalized against actin, in comparison with siCo. Significant *P*-values are indicated in the graphs **P<0.01, ***P<0.001.

Given that B-CLL lymphocytes exhibit apoptosis resistance, a hallmark of this disease, we sought to determine whether *NTSR2* downregulation would sensitize B-CLL cells to programmed cell death. To this end, we performed mRNA silencing assays with a pool of four different siRNAs directed against *NTSR2* mRNA (Figures 2a–f).

In the four patients tested, *NTSR2* silencing triggered a drastic decrease in cell viability, from 86 to 24%, after 72 h (Figure 2c, *P*<0.001). To extend these observations, we investigated the pro-apoptotic effect of NTSR2 silencing after 48 h using flow cytometry to detect Annexin V/PI (propidium iodide) staining (Figures 2d and e). *NTSR2* depletion significantly increased the percentage of Annexin V-positive cells, reflecting induction of apoptosis (*n*=4, *P*<0.01, Figures 2d and e). However, we observed no alternative cell death (Annexin V^negative^/PI^positive^) (Figure 2d). Further evidence for apoptosis was obtained by enzyme-linked immunosorbent assay (ELISA) to detect DNA fragmentation.^16^ Consistent with the results of flow cytometry, *NTSR2* depletion triggered DNA fragmentation, as demonstrated by elevated levels of cytoplasmic nucleosomes (*P*<0.01, Figure 2f) in comparison to control and isolated normal B cells (Supplementary Figures 1a–c).

In B-CLL pathogenesis, both survival signaling and anti-apoptotic proteins are often dysregulated (Supplementary Figures 1d–f). Accordingly, we investigated the expression of the Src pro-survival signaling pathways and the anti-apoptotic protein Bcl-2 following *NTSR2* depletion. Although Bcl-2 was highly expressed in B-CLL cells (Supplementary Figure 1f), its levels decreased significantly upon *NTSR2* depletion (Figures 2g and i). Likewise, the pro-survival kinase Src exhibited a decrease in constitutive phosphorylation (Figures 2g and h), suggesting that *NTSR2* makes a major contribution to Src kinase activation and expression of anti-apoptotic proteins.

**Figure 3:**
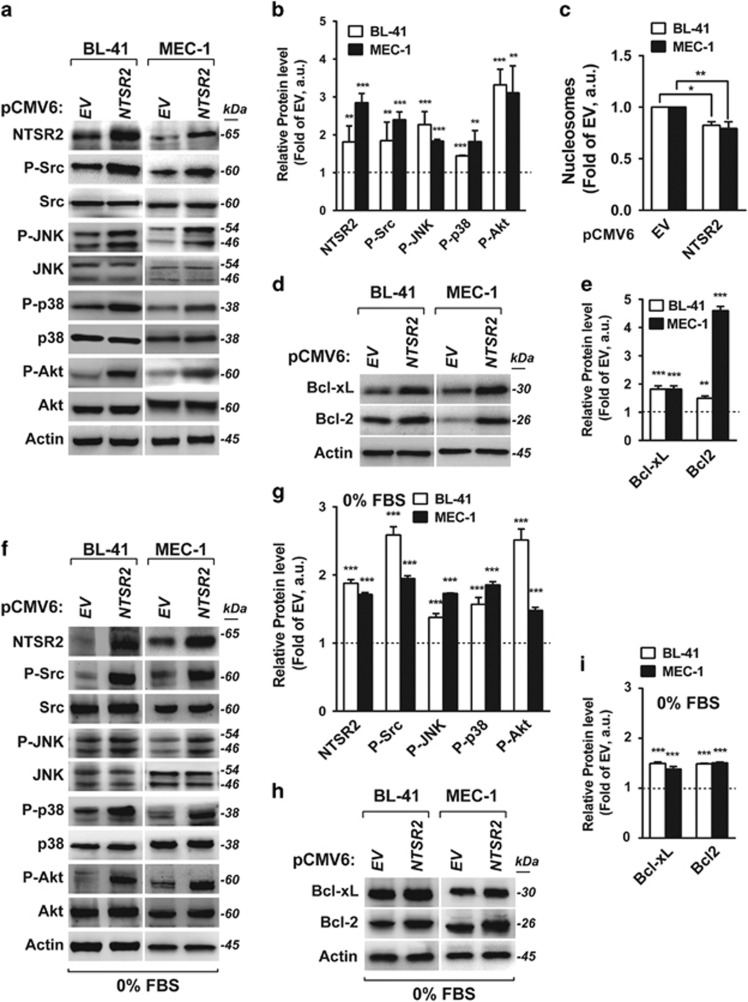
NTSR2 overexpression induces cell survival signaling pathways. (**a**,**b**) Western blot analysis of NTSR2, Src, *SAPK/JNK*, p38MAPK, and Akt expression from lysates of BL-41 and MEC-1 cells overexpressing NTSR2 (pCMV6-NTSR2) or empty vector (EV) cultured for 24 h post-transfection in basal conditions (10% fetal bovine serum). (**c**) Apoptotic ratio following NTSR2 overexpression for 24 h, assessed by cell death ELISA, expressed as fold change vs empty vector (EV). (**d**, **e**) Representative western blot analysis of Bcl-xL and Bcl-2 expression in BL-41 and MEC-1 cells overexpressing NTSR2 for 24 h. (**h**, **i**) Fold change in Bcl-xL or Bcl-2 expression in BL-41 and MEC-1 cells overexpressing NTSR2 for 24 h, normalized against actin, in comparison with EV. (**f–i**) Similar analyses performed in cells deprived of serum for 24 h. Significant *P*-values are indicated in the graphs *P<0.05, **P<0.01, ***P<0.001.

In the following experiments, we used two human B-lymphocyte cell lines, one from lymphoma (BL-41) and one from B-CLL (MEC-1). Although endogenous expression of NTSR2 was higher in BL-41 and MEC-1 than in normal B cells, it was lower than in B-CLL (Supplementary Figure 1g). Hence, to achieve comparable expression levels between models, we overexpressed NTSR2 in both BL-41 and MEC-1 cells (Supplementary Figure 2a). Because the NTSR2 signaling pathway is connected to three major MAPK pathways, that is, ERK1/2, p38 and JNK, as well as the PI3K/AKT survival pathway, we used arrays to monitor the presence of phosphorylated MAPKs following *NTSR2* overexpression (Supplementary Figure 2b). Strikingly, *NTSR2* overexpression resulted in a 2–3-fold induction (that is, increase in phosphorylation) of several proteins engaged in survival pathways, including Src, JNK, p38 and Akt (Figures 3a and b). In addition, we observed a decrease in the amount of cytoplasmic nucleosomes, reflecting the level of apoptosis (Figure 3c), following an increase in the expression of the anti-apoptotic proteins Bcl-xL and Bcl-2 (Figures 3d and e). Similar results were obtained when cells were deprived of serum for 24 h, ruling out the possibility that NTSR2 is activated by ligands present in calf serum (Figures 3f–i). Together, these results indicate that *NTSR2* overexpression acts as an oncogene via stimulation of the Src and MAP kinases, as well as by upregulating expression of anti-apoptotic proteins, thereby promoting cell survival and apoptosis resistance, respectively. These results reveal a specific effect of NTSR2 on pro-survival signaling in B-CLL cells.

### NTSR2 stimulation is independent of its natural ligand, NTS

**Figure 4:**
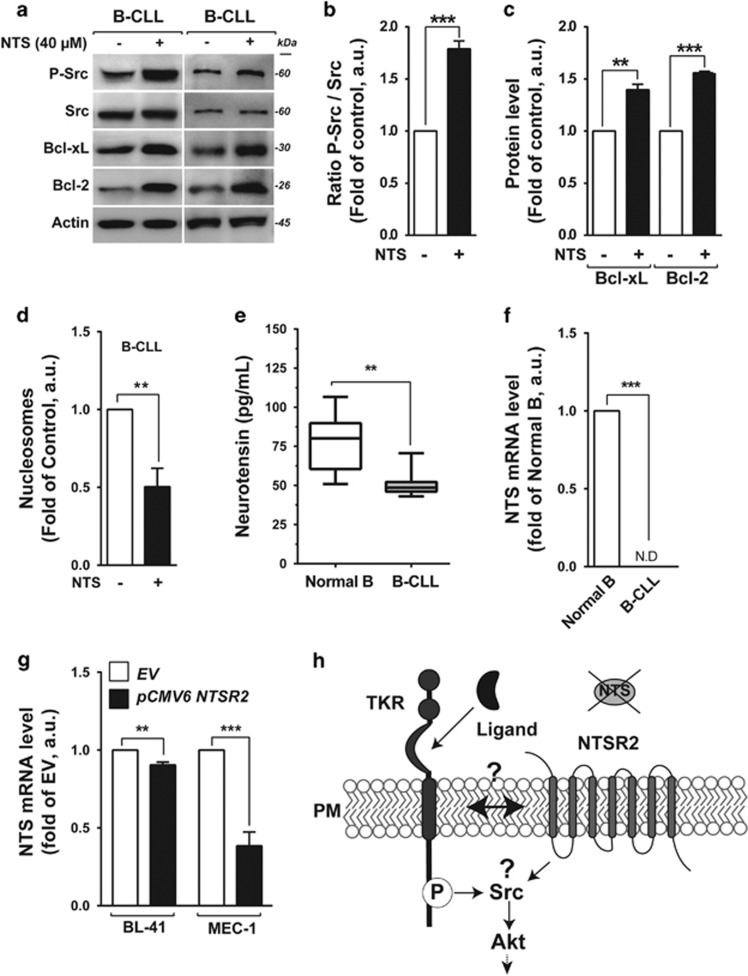
Exogenous neurotensin (NTS) maintains cell survival pathways in B-CLL. (**a**) Representative western blot of p-Src, Bcl-xL and Bcl-2 expression in B-CLL cells after addition of neurotensin (40 μM) for 24 h. (**b**,**c**) Expression levels of P-Src (**b**), Bcl-xL and Bcl-2 (**c**) represented as, respectively, the ratio of phosphorylated Src vs pan-Src protein and the ratios of Bcl-xL and Bcl-2 to actin. Values are means±s.e.m. of B-CLL, expressed in a.u. (*n*=3). (**d**) Apoptotic ratio in B-CLL in the presence or absence of 40 μM NTS for 24 h, assessed by cell death ELISA. Values are proportions of apoptotic cells (±s.e.m.) in three independent experiments from different patients (*n*=3). (**e**) Neurotensin concentration, quantified by ELISA, from B-CLL patient plasma (*n*=22, gray boxes), in comparison with healthy donor plasma (*n*=8, white boxes). (**f**) Quantitative analysis of *NTS* mRNA level in normal B cells (*n*=15) and B-CLL (*n*=30). Data are expressed as mean fold change in expression (±s.e.m.) in comparison with normal B cells. ND: not detectable. (**g**) Quantitative analysis of *NTS* mRNA level in BL-41 or MEC-1 transfected with either *NTSR2* expression vector (*p*CMV6 NTSR2) or empty vector (EV). Data are expressed as mean fold change (±s.e.m.) vs empty vector. (**h**) Schematic representation of hypothetical NTSR2 activation dependent on recruitment of a tyrosine kinase receptor (TKR). Significant *P*-values are indicated in the graphs **P<0.01, ***P<0.001.

We hypothesized that NTSR2 stimulation depended on activation of autocrine and/or paracrine loops following release of NTS, as we showed previously for NTSR1 in normal B lymphocytes.^14^ To investigate the role of NTS in modulating apoptosis, we monitored its effect on expression in B lymphocytes derived from B-CLL patients. As observed for NTSR1, the addition of NTS to B-CLL lymphocytes promoted sustained activation of NTSR2 and its downstream cascade, including phosphorylation of Src kinase (Figures 4a and b) and expression of the anti-apoptotic proteins Bcl-2 and Bcl-xL (Figures 4a and c). Following this stimulation, the apoptotic ratio of B-CLL cells decreased significantly (Figure 4d). Because NTSR1 was not expressed in B-CLL, these observations suggest that NTSR2 plays a protective role in these cells.

To determine whether the autocrine/paracrine interaction of the NTS–NTSR2 complex takes place in B-CLL patients, we performed ELISA to measure the concentration of circulating NTS in plasma from B-CLL patients and HDs. Surprisingly, the concentration of NTS was higher in HD than in B-CLL plasma (80.03 pg/ml vs 48.62* *pg/ml, *P*<0.01, Figure 4e). *NTS* mRNA was not detectable (ND) in B-CLL cells, in contrast to normal B cells (Figure 4f), excluding the possibility of autocrine and/or paracrine survival loops triggered by NTS–NTSR2 on the surface of B-CLL cells. Likewise, in both BL-41 and MEC-1 cell lines, overexpression of *NTSR2* for 24 h did not trigger *NTS* expression (Figure 4g), despite sustained NTSR2 activation irrespective of growth factor abundance (Figure 3f).

Taken together, these results suggested that NTSR2 activation must be sustained by an alternative mechanism that does not involve the canonical ligand NTS. We hypothesized (Figure 4h) that, as observed for NTSR1^12, 17^ and NTSR3/Sortilin,^18, 19^ NTSR2 can recruit a TKR as an alternative second messenger, thereby inducing its own activation.

### NTSR2 interacts with the oncoreceptor TrkB

We previously demonstrated interactions between NTSR3 and epidermal growth factor receptor (EGFR) and TrkB.^18^ Interestingly, NTSR1 also interacts with EGFR, although, as mentioned above, neither NTSR1 nor EGFR is detectably expressed in B-CLL cells.^20^ Based on our previous findings showing that TrkB and its ligand BDNF are involved in fine-tuning endogenous B-cell survival,^21^ as well as in several malignancies^22^ including myeloma,^23, 24^ we speculated that TrkB might serve as a co-receptor for NTSR2.

**Figure 5:**
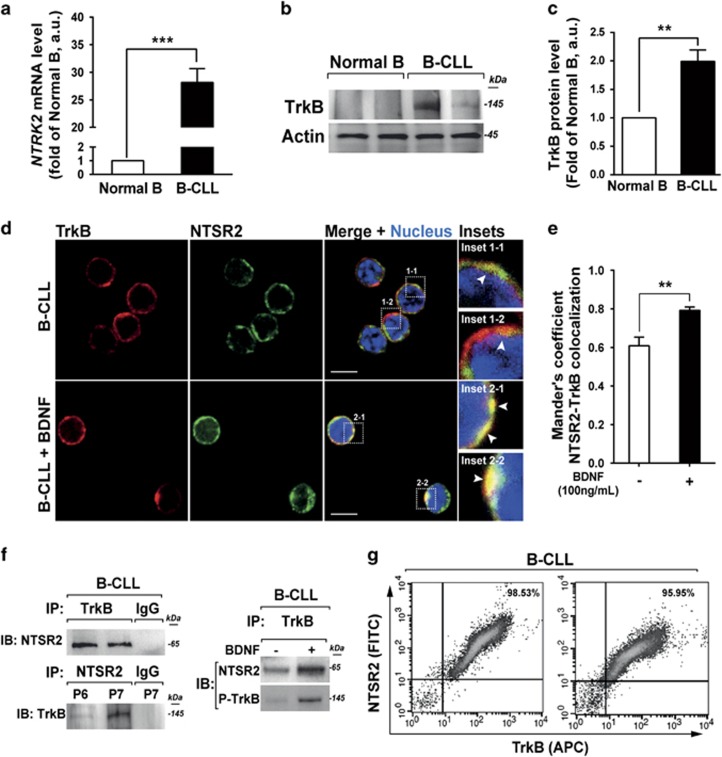
NTSR2 interacts with the tyrosine kinase receptor TrkB. (**a**) Quantitative analysis of the level of *NTRK2* mRNA (encoding TrkB) in normal B (*n*=15) and B-CLL (*n*=30) lymphocytes. Data are expressed as mean fold change in *NTRK2* expression (±s.e.m.) vs normal B cells. (**b**) Representative analysis of TrkB expression from B-lymphocyte cell lysates from two normal donors (D1, D2) or two B-CLL patients (P1, P2). (**c**) TrkB expression level, normalized against actin, in normal B (*n*=6) and B-CLL (*n*=6) cells. Values are means±s.e.m. of three independent experiments. (**d**) Confocal microscopy analysis of NTSR2 (green) and TrkB (red) and their colocalization in B-CLL cells (yellow staining in merged image, insets 1-1 to 2-2) in the presence or absence of BDNF (100 ng/ml). (**e**) Mander’s overlap coefficient indicating colocalization of NTSR2 and TrkB after BDNF (100 ng/ml) treatment (means±s.e.m. of three independent experiments). (**f**) After immunoprecipitation (IP) of TrkB and NTSR2 from B-CLL protein lysates, immunocomplexes were immunoblotted (IB) with the indicated antibodies. (**g**) Representative cytogram showing co-detection of NTSR2 and TrkB by flow cytometry in B-CLL lymphocytes. Significant *P*-values are indicated in the graphs **P<0.01, ***P<0.001.

To explore this possibility, we first compared the levels of *NTRK2* mRNA (encoding TrkB) in B lymphocytes between HDs (*n*=15) and B-CLL patients (*n*=30). *NTRK2* was expressed at 30-fold higher levels (*P*<0.001) in B-CLL cells than in normal B lymphocytes (Figure 5a). Analysis of TrkB expression at the protein level confirmed the mRNA results (Figures 5b and c), consistent with the notion that TrkB could act as a co-receptor of NTSR2.

Confocal analyses revealed that NTSR2 and TrkB colocalized on the B-CLL cell surface (Figure 5d, insets 1-1 and 1-2). Interestingly, this colocalization was more prominent following activation of TrkB by its ligand BDNF (Figure 5d, insets 2-1 and 2-2). These observations were also supported by colocalization analysis based on Mander’s overlap coefficient (Figure 5e). Immunoprecipitation (IP) demonstrated that NTSR2 interacted physically with TrkB in B-CLL cells, and this interaction was promoted upon BDNF stimulation, as demonstrated by the presence of phosphorylated TrkB (p-TrkB) in the NTSR2–TrkB immunocomplex (Figure 5f). These interactions were also observed in BL-41 and MEC-1 models (Supplementary Figure 3a) but were absent from normal B lymphocytes (data not shown), suggesting that the NTSR2–TrkB interaction plays a critical role in B-CLL cell homeostasis.

**Figure 6:**
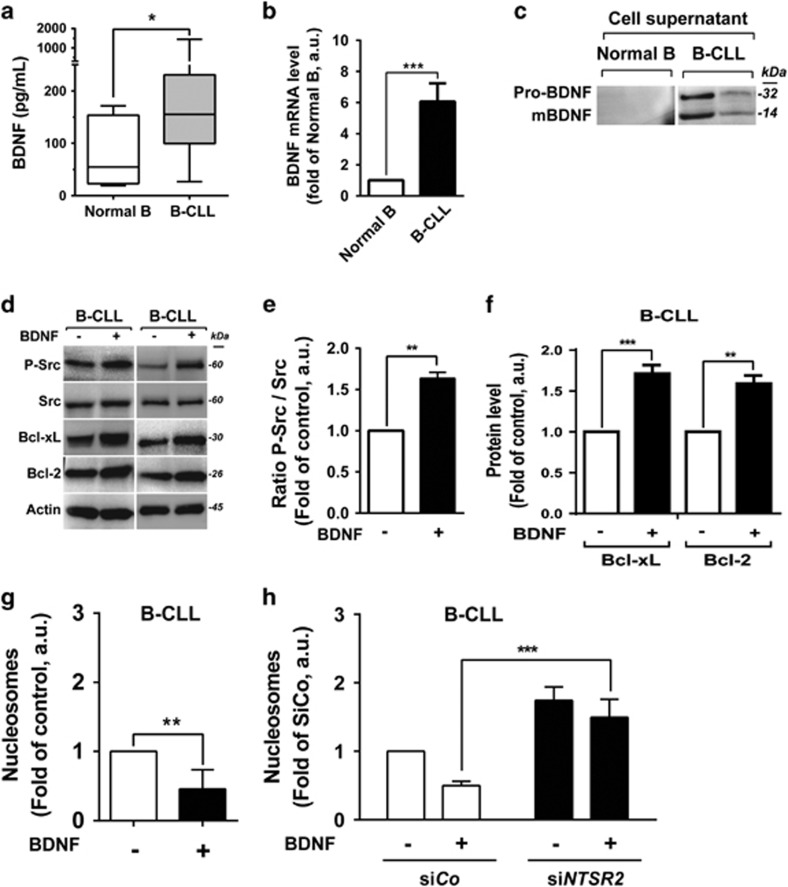
Protective role of BDNF against B-CLL apoptosis. (**a**) BDNF concentration, quantified by ELISA, in B-CLL patient plasma (*n*=17, gray boxes) in comparison with healthy donor plasma (*n*=9, white boxes). (**b**) Quantitative analysis of *BDNF* mRNA level in normal B (*n*=15) and B-CLL (*n*=30) cells. Data are expressed as mean fold change in expression (±s.e.m.) vs normal B cells. (**c**) Representative western blot analysis of pro-BDNF and mature BDNF (mBDNF) expression in normal B and B-CLL supernatants after 24 h of culture post-isolation. (**d**) Western blot analysis of P-Src, Bcl-xL and Bcl-2 in B-CLL cell lysates from two patients after addition of BDNF (100 ng/ml) for 24 h. (**e**, **f**) Expression levels of p-Src (**e**), Bcl-xL and Bcl-2 (**f**) represented as, respectively, the ratio of phosphorylated Src vs pan-Src protein and the ratios of Bcl-xL and Bcl-2 to actin (B-CLL, *n*=3). Values are means±s.e.m. of three independent experiments, in a.u. (**g**, **h**) Apoptotic ratio, assessed by cell death ELISA, in B-CLL cells cultured with or without 100 ng/ml BDNF for 24 h (B-CLL, *n*=4) in the presence or absence of siRNA against NTSR2 or control siRNA. Values are the mean ratio of apoptotic cells (±s.e.m.) from four independent experiments. Significant *P*-values are indicated in the graphs *P<0.05, **P<0.01, ***P<0.001.

Because BDNF stimulation promoted formation of the NTSR2–TrkB complex in leukemia cells, we analyzed both BDNF expression in B-CLL and circulating BDNF in plasma derived from B-CLL patients or HDs. In contrast to NTS, the concentration of BDNF was higher in B-CLL than in HD plasma (155.2 pg/ml vs 54.58* *pg/ml, *P*<0.01, Figure 6a). Likewise, *BDNF* transcript levels were significantly higher in B-CLL than in normal B cells (Figure 6b). Consistent with this, both mature BDNF (mBDNF) and its precursor form (pro-BDNF) were detected in the supernatants of isolated B-CLL lymphocytes, but not normal B cells (Figure 6c), suggesting activation of autocrine and/or paracrine survival loops. As with NTS, the addition of BDNF to B-CLL lymphocytes promoted sustained activation of NTSR2 and its downstream cascade, including phosphorylation of Src kinase and expression of the anti-apoptotic proteins Bcl-2 and Bcl-xL (Figures 6d–f). Likewise, upon TrkB inhibition, NTSR2 failed to maintain both Src Kinases phosphorylation and anti-apoptotic proteins expression thereby suggesting that TrkB and NTSR2 contribute together in the pro-survival signaling pathway (Supplementary Figures 4a–c).

In addition, *in vitro* experiments performed on B-CLL cells following BDNF stimulation revealed a reduced apoptotic ratio in B-CLL, suggesting a protective role for the NTSR2–TrkB–BDNF complex (Figure 6g). To rule out the possibility that the BDNF–TrkB interaction stimulates an autocrine survival loop independently of NTSR2, we performed *NTSR2* silencing prior to BDNF stimulation. BDNF failed to protect B-CLL cells from apoptosis triggered by *NTSR2* inactivation, suggesting that TrkB is a potential driver for B-CLL survival only when *NTSR2* is overexpressed (Figure 6h).

In both MEC-1 and BL-41 cells, *NTSR2* overexpression triggered *NTRK2* expression (Supplementary Figure 3b). Likewise, in cells overexpressing *NTRK2*, we observed an increase in the level of *NTSR2* transcripts (Supplementary Figure 3c). These observations are supported by the flow cytometry results demonstrating that NTSR2 and TrkB are co-expressed with at similar levels by B-CLL cells (Figure 5g). Together, these findings indicate that expression of NTSR2 in leukemic patients results from *NTRK2* overexpression, and vice versa, providing a molecular mechanism that could explain *NTSR2* overexpression in B-CLL.

Overall, these observations suggested that the NTSR2–TrkB complex is at the center of a regulatory network in B-CLL and acts as a conditional oncogene in the presence of BDNF.

### NTSR2 is constitutively phosphorylated both in B-CLL cells and model cell lines

**Figure 7:**
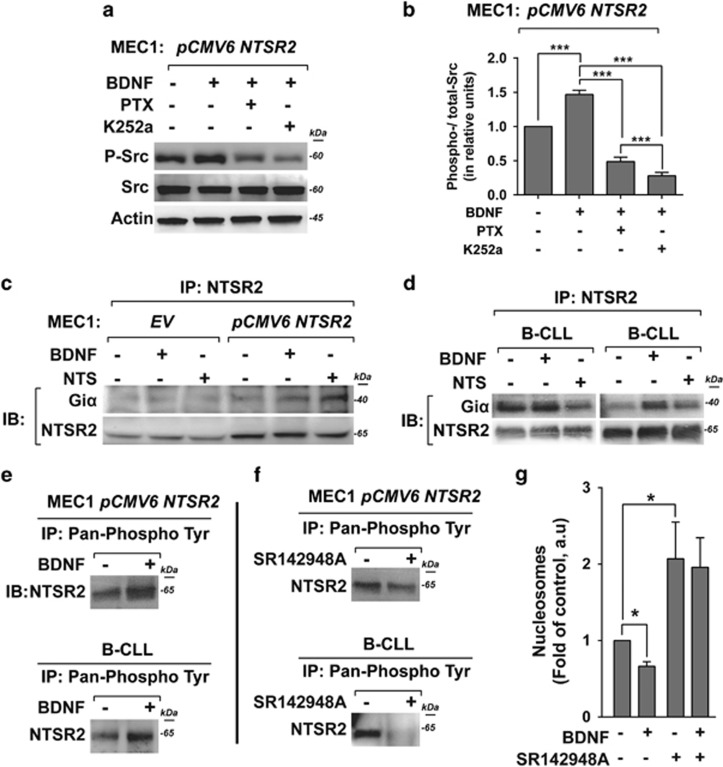
NTSR2 phosphorylation in B-CLL and recruitment of G_i_α proteins. (**a**) Representative western blot analysis of Src phosphorylation in MEC-1 cells overexpressing NTSR2 (pCMV6-NTSR2) in the presence or absence of BDNF (100 ng/ml), pertussis toxin (PTX, 200 ng/ml) or K252a (100 nM) for 24 h. (**b**) Ratio of phosphorylated Src vs pan-Src protein, normalized against actin. Values are means±s.e.m. of three independent experiments in a.u. (**c**, **d**). After immunoprecipitation (IP) of NTSR2 from MEC-1 cells overexpressing NTSR2 (pCMV6-NTSR2) or not (EV), or from B-CLL cells in the presence or absence of BDNF (100 ng/ml) or NTS (40 μM/ml), the immunocomplexes were immunoblotted (IB) with anti-G_i_α1/2 antibodies. (**e**, **f**) After immunoprecipitation (IP) of anti-pan-phosphoprotein was performed on MEC-1 cells overexpressing NTSR2 and B-CLL cells in the presence or absence of BDNF (100 ng/ml) or SR142948A (67 μM), the phosphorylation of NTSR2 was detected by immunoblotting (IB) with anti-NTSR2 antibodies. (**g**) Apoptotic ratio, assessed by cell death ELISA, in B-CLL in the presence or absence of SR142948A (67 μM) for 24 h. Values are mean ratios of apoptotic cells (±s.e.m.) of three independent experiments from different patients (*n*=3). Significant *P*-values are indicated in the graphs *P<0.05, ***P<0.001.

Next, we sought to characterize the activation of NTSR2 through the recruitment of G_i_α proteins, which have been implicated in the activities of several GPCRs such as those observed in immune cell activation.^5^ To this end, we studied the effect of pertussis toxin (PTX), which interferes with the interaction of Gα protein subunits of the G_i_ family (isoforms G_i_α1, G_i_α2 and G_i_α3).^5^ Interestingly, upon PTX treatment, BDNF stimulation failed to maintain Src phosphorylation in NTSR2-overexpressing MEC-1 cells (Figures 7a and b). Likewise, Src phosphorylation was reduced in the presence of K252a, a TrkB inhibitor (Figures 7a and b), suggesting that activation of Src upon BDNF stimulation depends on G-protein recruitment and TrkB activation. Accordingly, we next investigated the ability of NTSR2 to recruit G_i_α proteins upon stimulation with BDNF, in comparison with its natural ligand NTS. Indeed, IP performed on MEC-1 cells overexpressing NTSR2 indicated that G_i_α 1/2 proteins interacted physically with NTSR2, and that this interaction was strengthened upon BDNF stimulation, as also observed upon NTS treatment (Figure 7c), suggesting that BDNF can trigger recruitment of G_i_α 1/2. In addition, we detected a physical interaction between G_i_α 1/2 and NTSR2 in isolated B-CLL lymphocytes (Figure 7d). In the B-CLL, G_i_α 1/2 was recruited more efficiently after stimulation with BDNF than with NTS, probably reflecting a stronger tropism for BDNF, which is more highly expressed than NTS in B-CLL cells (Figure 4f). Likewise, in the absence of stimulation, the first patient exhibited strong recruitment of G_i_α 1/2, suggesting a potential difference in the concentration of mBDNF produced and released by B-CLL cells, as observed previously (Figure 6c). Together, these results suggest that NTSR2 undergoes modifications to sustain its signaling pathways and recruit G-protein subunits.

Given that phosphorylation of NTSR2 was previously observed in mouse^25^ and in human,^26^ we investigated whether NTSR2 is phosphorylated in NTSR2-OE cell lines and human B-CLL lymphocytes. We detected NTSR2 phosphorylation in MEC-1 cells overexpressing NTSR2, as well as in B-CLL lymphocytes, and the phosphorylation levels increased upon BDNF stimulation (Figure 7e). In the presence of SR142948A, a competitive inhibitor of NTSR ligand binding, NTSR2 phosphorylation was suppressed, supporting the binding of BDNF (Figure 7f). This result agreed with the increase of apoptosis observed in B-CLL exposed to SR142948A (Figure 7g), suggesting that NTSR2 phosphorylation is crucial for B-CLL cell survival.

Taken together, our findings show that NTSR2 overexpression induces Giα recruitment and NTSR2 phosphorylation in B-CLL lymphocytes. The colocalization of NTSR2 with TrkB, which is promoted by the presence of BDNF, indicates that a GPCR signaling platform including both receptors is associated with apoptosis resistance in B-CLL pathogenesis.

## Discussion

Resistance to apoptosis is a hallmark of B-CLL clonal lymphocytes, which are characterized by elevated expression of anti-apoptotic Bcl-2 family proteins^27^ and sustained activation of pro-survival signaling pathways.^28, 29^ Several studies have reported various mechanisms associated with B-CLL cell survival, many of which are related to B-cell receptor (BCR) activation, However, despite encouraging results using kinase inhibitors targeting the BCR pathway,^30^ the frequency of resistance to this treatment and relapse in CLL patients highlights the deficiencies of current treatment protocols and suggests that other mechanisms are involved in CLL pathogenesis.

**Figure 8:**
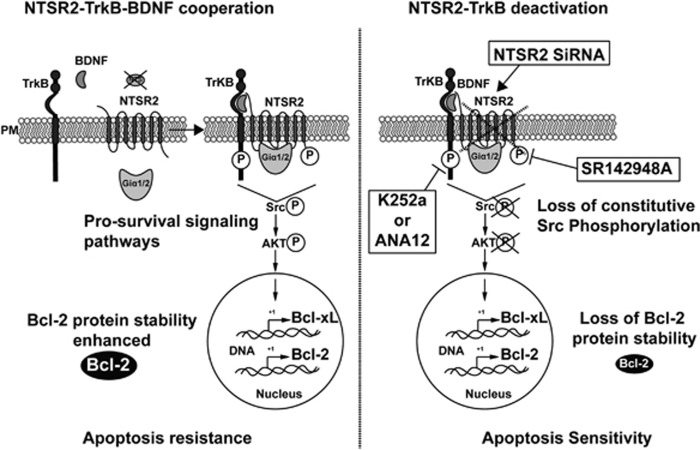
Model of NTSR2 function in B-CLL survival. In this schematic, NTSR2–TrkB–BDNF acts as a key regulator of B-CLL resistance to apoptosis. The NTSR2–TrkB interaction is strengthened upon BDNF stimulation, and triggers pro-survival pathways via phosphorylation of NTSR2, resulting in activation of the phosphorylation cascade of the Src and AKT kinases, as well as expression of the downstream anti-apoptotic proteins Bcl-xL and Bcl-2, leading to B-CLL cell survival and resistance to apoptosis (left panel). NTSR2 inhibition by siRNA-mediated mRNA depletion induces a drastic apoptotic cell death despite the presence of TrkB and BDNF, indicating that TrkB plays a role as a second messenger in NTSR2-mediated apoptotic resistance in B-CLL. NTSR2 deactivation by SR142948A suppresses the ability of NTSR2 to recruit the Giα1/2 subunits upon BDNF stimulation, leading to the suppression of NTSR2 phosphorylation and a decrease in the expression of anti-apoptotic proteins, thereby increasing B-CLL apoptosis (right panel). PM, plasma membrane.

Independently of BCR activation, several pro-survival B-CLL growth factors and non-BCR receptors have been implicated in the pathogenesis of B-CLL,^31^ including Notch,^32^ TKR receptors such as insulin-like growth factor-1 receptor,^33, 34^ and chemokines and their associated GPCRs.^35, 36, 37, 38, 39^ However, other GPCRs have not previously been reported to be involved in B-CLL pathogenesis. In this report, we provide the first evidence for the role of another GPCR, NTSR2, in B-CLL resistance to apoptosis (Figure 8). These data are consistent with our previous results showing that NTSR1 and NTSR2 and their common ligand NTS are involved in fine-tuned regulation of normal B-lymphocyte survival.^14^ However, in B-CLL lymphocytes, NTSR2 was drastically overexpressed, whereas NTS and NTSR1 were strongly downregulated. Therefore, we investigated the significance of NTSR2 overexpression in the absence of its ligand in the context of B-CLL lymphocyte survival. Our findings revealed that NTSR2 inactivation by either siRNA or a pharmacological inhibitor re-established B-CLL apoptosis (Figure 8, right panel). NTSR2 inactivation was associated with a significant decrease in the levels of the anti-apoptotic protein Bcl-2, along with reduced activation of Src, which is constitutively phosphorylated in B cells from CLL patients. Strikingly, whereas Bcl-2 protein level changed following NTSR2 stimulation or inhibition, its mRNA level remains unchanged suggesting that Bcl-2 protein undergoes post-translational modifications increasing thereby its stability^40, 41, 42^ (Supplementary Figure 5). These observations will need to be resolved more deeply in future studies

Because NTS was undetectable in B-CLL lymphocytes, we investigated the possibility that a cross-pathway interaction with another partner was responsible for activating B-CLL cell survival. Like NTS, the growth factor BDNF, a member of the neurotrophin family, is known to participate in B-cell apoptosis resistance via TrkB, its TKR.^21^ The concomitant effects of TrkB and NTSR2 on B-CLL lymphocyte survival depended on a physical interaction between these receptors, which was strengthened by BDNF activation (Figure 8, left panel). No such interaction was detected in normal B lymphocytes. This interaction was supported by mutual regulation of these receptors at the transcriptional level. TrkB expression was upregulated in B-CLL cells, as well as in NTSR2-overexpressing BL-41 or MEC-1 cells. Moreover, experimental overexpression of TrkB in MEC-1 and BL-41 cells induced the upregulation of *NTSR2* mRNA. A cross-pathway interaction between GPCRs and TKRs was previously reported for another NTS receptor, NTSR1 and EGFR/HERs in lung, breast and hepatocarcinomas.^12, 17, 43^ In leukemias or lymphomas, NTSR1 has been characterized in Sezary syndrome and HL-60 cells,^44, 45^ whereas NTSR2 has rarely been investigated in cancers, with the exception of prostatic cancer.^46^

In this study, we showed that B-CLL survival is linked to the activation of NTSR2, as assessed by G-protein recruitment and phosphorylation (Figure 8, left panel). NTSR2 activation depends on G_i_α protein, as demonstrated by two observations: G_i_α proteins immunoprecipitate with NTSR2 receptor in lymphocytes from B-CLL patients, and PTX decreases cell activation in NTSR2-overexpressing cells. G_i_α protein recruitment in immune cells plays a central role in B-lymphocyte survival, especially in regard to control of Bcl-2 expression, as demonstrated in G_i_α-deficient mouse models showing that downregulated expression of Bcl-2 family proteins.^47^ That, decreases the B-1a B-lymphocyte subpopulation,^48^ equivalent to B-CLL lymphocytes. However, NTSR has not been previously examined with regard to B-CLL survival. This function was assessed by examining the recruitment and activation of Src, which is constitutively activated in B-CLL lymphocytes. The inhibition of NTSR by either a NTSR pharmacological inhibitor or by si*NTSR2* in B-CLL lymphocytes restored B-CLL apoptosis.

According to other TKR–GPCR platform studies,^49, 50, 51, 52, 53^ NTSR2 activation is associated with the TrkB–NTSR2 complex, an active TKR–GPCR complex that can activate pro-survival signaling pathways including p38MAPK, Erk1/2, JNK and Src. Activation of signaling pathways following BDNF treatment suggests that NTSR2 is activated by TrkB, a finding not previously reported. A TKR–GPCR interaction leading to downstream activation through G_i_α recruitment was reported for another TKR receptor, IGFR, and another GPCR, CCR5 activated by a chemokine.^52^ TKR/GPCR complex involving neurotrophin receptors was also previously reported for TrkA interacting with LPA (lysophosphatidate receptor) a GPCR receptor, activated by the TrkA ligand NGF (nerve growth factor), whereas GPCR inhibition causes a decrease in MAPK signaling.^54, 55^

The relationship between anti-apoptotic protein expression, resistance to apoptosis in B-CLL, and NTSR2 overexpression reveals a new mechanism of aberrant B-CLL lymphocyte survival. Knowledge of this mechanism could support the targeting of NTSR2 and its activation, with the ultimate goal of clinical application.

## Materials and methods

### Cell cultures

BL-41 and MEC-1 cell lines (Leibniz Institute, DSMZ, Germany) were cultured as previously described.^14^ Human B lymphocytes from healthy donors blood samples, obtained after approval of the Institutional Ethic Committee in accordance with the Declaration of Helsinki, were isolated as previously reported.^14, 21^ Thirty-four B-CLL patients were included in this study and approved by Institutional Review Board AC 72-2011-18. B-CLL were isolated from venous blood using the MACSxpress B-CLL Cell Isolation Kit, human (MACS; Miltenyi Biotec, Bergisch Gladbach, Germany) according to the manufacturer’s instructions.

### Overexpression and RNA interference

cDNAs encoding NTSR2 and TrkB in pCMV6-XL4 expression vectors were purchased from OriGene Technologies (Herford, Germany). For transient overexpression, cells were transfected using Amaxa Nucleofector 2b (Lonza, Levallois-Perret, France) and the Amaxa Nucleofector Kit-V (Lonza) according to the manufacturer’s instructions. For interference assays, B-CLL cells were transfected using INTERFERin (Polyplus transfection, Illkirch, France). In each transfection, 84 ng siRNA against *NTSR2* (ON-TARGETplus Human *NTSR2* [23620] siRNA – SMARTpool), or siRNA control (ON-TARGETplus Non-targeting pool D-001810-10-20) (Dharmacon, CO, USA), were used.

### Drugs and treatments

Cultured cells were incubated for 24 h with 40 μM NTS (Calbiochem/Merck Millipore, Fontenay sous Bois, France) or 100 ng/ml human recombinant BDNF (Alomone labs, Jerusalem, Israel). Optimal concentrations of exogenous BDNF and NTS were determined previously.^14, 21^ The non-peptide NTSR antagonist SR142948A (Tocris Biosciences, Bristol, UK) was used at 67 nM,^56^ PTX (Sigma-Aldrich, St Louis, MO, USA) at 200 ng/ml^35, 57^; TrkB inhibitors were K252a (Alomone Labs) at 100 nM^58, 59^ or ANA12 (Tocris Biosciences) at 100 μM.^60^

### Flow cytometry and immunofluorescence

Cells were fixed in 4% paraformaldehyde in phosphate buffered saline (PBS) for 15 min at room temperature (RT). After washes in 1% bovine serum albumin/PBS and incubation with 5% bovine serum albumin/PBS for 30 min at RT, cells were incubated overnight at 4 °C with both rabbit polyclonal anti-NTSR2 and mouse anti-TrkB diluted in PBS/1% bovine serum albumin. After washing, samples were incubated with 1 mg/ml Alexa Fluor 488- or 594-conjugated anti-rabbit or anti-mouse IgG Ab (Invitrogen, Carlsbad, CA, USA) for 1 h at RT. After washes, cells were suspended in 500 μl of PBS and analyzed on a FACSCalibur flow cytometer (Becton Dickinson, Heidelberg, Germany) mounted in mounting medium containing 4',6-diamidino-2-phénylindole (DAPI) (Sigma-Aldrich) and observed under a confocal microscope (Carl Zeiss, LSM 880, Oberkochen Germany).

### Western blots

Proteins obtained as described previously^58^ were blotted onto polyvinylidene fluoride membrane (Calbiochem/Merck Millipore) and incubated with following antibodies: rabbit anti-NTSR-1 (ANT-015), rabbit anti-NTSR-2 (ANT-016), Alomone Labs; rabbit anti-Bcl-2 (sc-783, Santa Cruz Biotechnology, Dallas, TX, USA); rabbit anti-Bcl-xL (#2764S), rabbit anti-phospho-Akt (Ser473) (#4060S), mouse anti-Akt (pan; #2920S), rabbit anti-phospho-Src family (Tyr416; #6943S), rabbit anti-Src (#2108S), rabbit anti-phospho-p38 MAPK (Thr180/Tyr182; #9211S), rabbit anti-p38 MAPK (#8690S), rabbit anti-phospho-SAPK/JNK (Thr183/Tyr185; #4668S), and rabbit anti-SAPK/JNK (#9252), all from Cell Signaling Technology (Danvers, MA, USA); mouse anti-Giα1/2 antibody (06-236, Calbiochem/Merck Millipore); and anti-actin (A5441, Sigma-Aldrich). After washing (tris-buffered saline/0.1% Tween-20), the immunoreactions were detected by incubation for 2 h at RT with horseradish peroxidase-conjugated secondary Ab against mouse or rabbit Ig (P0447 and P0448, respectively, Agilent Technologies, Santa Clara, CA, USA), revealed with the Immobilon Western Chemiluminescent HRP Substrate (Merck Millipore). Western blot were detected using Bioimaging Systems (GeneSnap and GeneTool; Syngene, Cambridge, UK). Densitometric analyses were performed using the ImageJ software (NIH, Bethesda, MD, USA).

### Immunoprecipitation

Immunoprecipitation were conducted as described previously.^58^ For IP assays, rabbit anti-NTSR-2 (ANT-016, Alomone Labs), mouse anti-TrkB (MAB3971, R&D Systems, Minneapolis, MN, USA), and mouse anti-phosphotyrosine antibody clone 4G10 (05-321, Calbiochem/Merck Millipore) were used along with 50 μl of protein A Sepharose (Sigma-Aldrich), and samples were incubated overnight at 4 °C. After centrifugation, the pellets were washed three times with IP buffer,^58^ and then resuspended in 30 μl of SDS–PAGE sample buffer containing DTT (50 mM) and analyzed by SDS–PAGE as described above for western blotting.

### Analysis of cell viability and apoptosis

Cell viability was assessed using Trypan Blue exclusion method.^61^ Apoptosis was evaluated using either the PI/Annexin V–fluorescein isothiocyanate double staining method as described previously^62^ or the Cell Death Detection ELISA PLUS kit (Roche, Basel, Switzerland) colorimetric assay according to the manufacturer’s instructions. Briefly, cells were transfected either with plasmids or SiRNA as described above and cultured with or without exogenous BDNF or NTS for 24 additional hours in a 96-multiwell plate (5 × 10^4^ cells/well). Absorbance values were measured at 405–490 nm with an ELISA reader (Thermo Fisher Scientific, Waltham, MA, USA). The absorbance obtained in controls was normalized to a value of 1, as previously described.^16^

### RNA extraction, reverse transcription and real-time quantitative PCR

RNA extraction, reverse transcription and real-time quantitative PCR analyses were performed as previously described.^14^ Primers and probes sequences used in this study are summarized in Table 1.

### Plasma NTS and BDNF quantification

Plasma BDNF and NTS levels were measured using commercial ELISA kits: BDNF E_max_ ELISA ImmunoAssay System (Promega, WI, USA) and Human Neurotensin, NT ELISA Kit (CUSABIO Life Science, College Park, MD, USA). All assays were performed in triplicate, and results are expressed in pg/ml.

### Data treatments and statistical analysis

Results were analyzed by one-way ANOVA followed by Fisher’s *post hoc* test using the StatView 5.0 software (Abacus Concepts, Piscataway, NJ, USA). *P*-values <0.05 were considered significant. Mean and s.e.m. values were obtained from at least three independent experiments.

## Figures and Tables

**Table 1:** Primers and probes sequences

*Names*	*Sequences*		*Length (pb)*	*GenBank Reference*
BDNF	Forward	5′-GGCTATGTGGAGTTGGCATT-3′	123	NM_170735.5
	Reverse	5′-CAAAACGAAGGCCTCTGAAG-3′		
	Probe	5′-ATTTCTGAGTGGCCATCCCAAGGTCTAG-3′		
Neurotensin	Forward	5′-TGACCAATATGCATACATCAAAGA-3′	105	NM_006183.4
	Reverse	5′-TAATTTGAACAGCCCAGCTG-3′		
	Probe	5′-CATGTTCCCTCTTGGAAGATGACTCTGCTA-3′		
NTSR1	Forward	5′-CGCCTCATGTTCTGCTACAT-3′	113	NM_002531.2
	Reverse	5′-TACGTCAGCTCCACCATCAA-3′		
	Probe	5′-AGCAGTGGACTCCGTTCCTCTATGACTTCT-3′		
NTSR2	Forward	5′-ATCCAGGTGAATGTGCTGGT-3′	114	NM_012344.3
	Reverse	5′-CCAAGTGCCGTCCACTTCTA-3′		
	Probe	5′-ACTAACTGCTTTCCTGAATGGGGTCACAGT-3′		
TrkB-FL	Forward	5′-CTGGTGAAAATCGGGGACT-3′	137	NM_006180.4
	Reverse	5′-AGGAAATTCACGACGGAAAG-3′		
	Probe	5′-TGTACAGCACTGACTACTACAGGGTCGGTG-3′		
BCL2	Applied Biosystems TaqMan Gene Expression Assay/Catalogue number 4331182		81	NM_000633.2
HPRT	HPRT1 Control mix, Applied Biosystems, TaqMan, VIC Catalogue number 4326321E			NM_000194.1

## References

[bib1] Kitada S, Pedersen IM, Schimmer AD, Reed JC. Dysregulation of apoptosis genes in hematopoietic malignancies. Oncogene 2002; 21: 3459–3474. 12032782 10.1038/sj.onc.1205327

[bib2] Chiorazzi N, Rai KR, Ferrarini M. Chronic lymphocytic leukemia. N Engl J Med 2005; 352: 804–815. 15728813 10.1056/NEJMra041720

[bib3] Rossi D, Rasi S, Spina V, Bruscaggin A, Monti S, Ciardullo C et al. Integrated mutational and cytogenetic analysis identifies new prognostic subgroups in chronic lymphocytic leukemia. Blood 2013; 121: 1403–1412. 23243274 10.1182/blood-2012-09-458265 PMC3578955

[bib4] Liu Y, An S, Ward R, Yang Y, Guo X-X, Li W et al. G protein-coupled receptors as promising cancer targets. Cancer Lett 2016; 376: 226–239. 27000991 10.1016/j.canlet.2016.03.031

[bib5] Kehrl JH. Heterotrimeric G protein signaling: roles in immune function and fine-tuning by RGS proteins. Immunity 1998; 8: 1–10. 9462506 10.1016/s1074-7613(00)80453-7

[bib6] Kehrl JH. The impact of RGS and other G-protein regulatory proteins on Gαi-mediated signaling in immunity. Biochem Pharmacol 2016; 114: 40–52. 27071343 10.1016/j.bcp.2016.04.005 PMC4993105

[bib7] Barragán M, Bellosillo B, Campàs C, Colomer D, Pons G, Gil J. Involvement of protein kinase C and phosphatidylinositol 3-kinase pathways in the survival of B-cell chronic lymphocytic leukemia cells. Blood 2002; 99: 2969–2976. 11929788 10.1182/blood.v99.8.2969

[bib8] Cuní S, Pérez-Aciego P, Pérez-Chacón G, Vargas JA, Sánchez A, Martín-Saavedra FM et al. A sustained activation of PI3K/NF-kappaB pathway is critical for the survival of chronic lymphocytic leukemia B cells. Leukemia 2004; 18: 1391–1400. 15175625 10.1038/sj.leu.2403398

[bib9] Dorsam RT, Gutkind JS. G-protein-coupled receptors and cancer. Nat Rev Cancer 2007; 7: 79–94. 17251915 10.1038/nrc2069

[bib10] Mazella J, Zsürger N, Navarro V, Chabry J, Kaghad M, Caput D et al. The 100-kDa neurotensin receptor is gp95/sortilin, a non-G-protein-coupled receptor. J Biol Chem 1998; 273: 26273–26276. 9756851 10.1074/jbc.273.41.26273

[bib11] Somaï S, Gompel A, Rostène W, Forgez P. Neurotensin counteracts apoptosis in breast cancer cells. Biochem Biophys Res Commun 2002; 295: 482–488. 12150975 10.1016/s0006-291x(02)00703-9

[bib12] Younes M, Wu Z, Dupouy S, Lupo AM, Mourra N, Takahashi T et al. Neurotensin (NTS) and its receptor (NTSR1) causes EGFR, HER2 and HER3 over-expression and their autocrine/paracrine activation in lung tumors, confirming responsiveness to erlotinib. Oncotarget 2014; 5: 8252–8269. 25249545 10.18632/oncotarget.1633 PMC4226681

[bib13] Dupouy S, Mourra N, Doan VK, Gompel A, Alifano M, Forgez P. The potential use of the neurotensin high affinity receptor 1 as a biomarker for cancer progression and as a component of personalized medicine in selective cancers. Biochimie 2011; 93: 1369–1378. 21605619 10.1016/j.biochi.2011.04.024

[bib14] Saada S, Marget P, Fauchais A-L, Lise M-C, Chemin G, Sindou P et al. Differential expression of neurotensin and specific receptors, NTSR1 and NTSR2, in normal and malignant human B lymphocytes. J Immunol 2012; 189: 5293–5303. 23109725 10.4049/jimmunol.1102937

[bib15] Bamford S, Dawson E, Forbes S, Clements J, Pettett R, Dogan A et al. The COSMIC (Catalogue of Somatic Mutations in Cancer) database and website. Br J Cancer 2004; 91: 355–358. 15188009 10.1038/sj.bjc.6601894 PMC2409828

[bib16] Frade JM, Rodríguez-Tébar A, Barde YA. Induction of cell death by endogenous nerve growth factor through its p75 receptor. Nature 1996; 383: 166–168. 8774880 10.1038/383166a0

[bib17] Dupouy S, Doan VK, Wu Z, Mourra N, Liu J, De Wever O et al. Activation of EGFR, HER2 and HER3 by neurotensin/neurotensin receptor 1 renders breast tumors aggressive yet highly responsive to lapatinib and metformin in mice. Oncotarget 2014; 5: 8235–8251. 25249538 10.18632/oncotarget.1632 PMC4226680

[bib18] Wilson CM, Naves T, Vincent F, Melloni B, Bonnaud F, Lalloué F et al. Sortilin mediates the release and transfer of exosomes in concert with two tyrosine kinase receptors. J Cell Sci 2014; 127: 3983–3997. 25037567 10.1242/jcs.149336

[bib19] Wilson CM, Naves T, Saada S, Pinet S, Vincent F, Lalloué F et al. The implications of sortilin/vps10p domain receptors in neurological and human diseases. CNS Neurol Disord Drug Targets 2014; 13: 1354–1365. 25345507 10.2174/1871527313666141023151642

[bib20] Dielschneider RF, Xiao W, Yoon J-Y, Noh E, Banerji V, Li H et al. Gefitinib targets ZAP-70-expressing chronic lymphocytic leukemia cells and inhibits B-cell receptor signaling. Cell Death Dis 2014; 5: e1439. 25275600 10.1038/cddis.2014.391 PMC4649506

[bib21] Fauchais A-L, Lalloué F, Lise M-C, Boumediene A, Preud’homme J-L, Vidal E et al. Role of endogenous brain-derived neurotrophic factor and sortilin in B cell survival. J Immunol 2008; 181: 3027–3038. 18713973 10.4049/jimmunol.181.5.3027

[bib22] Thiele CJ, Li Z, McKee AE. On Trk—the TrkB signal transduction pathway is an increasingly important target in cancer biology. Clin Cancer Res Off J Am Assoc Cancer Res 2009; 15: 5962–5967. 10.1158/1078-0432.CCR-08-0651 PMC2756331 19755385

[bib23] Pearse RN, Swendeman SL, Li Y, Rafii D, Hempstead BL. A neurotrophin axis in myeloma: TrkB and BDNF promote tumor-cell survival. Blood 2005; 105: 4429–4436. 15657181 10.1182/blood-2004-08-3096

[bib24] Ai L-S, Sun C-Y, Wang Y-D, Zhang L, Chu Z-B, Qin Y et al. Gene silencing of the BDNF/TrkB axis in multiple myeloma blocks bone destruction and tumor burden *in vitro* and *in vivo*. Int J Cancer 2013; 133: 1074–1084. 23420490 10.1002/ijc.28116

[bib25] Martin S, Vincent J-P, Mazella J. Recycling ability of the mouse and the human neurotensin type 2 receptors depends on a single tyrosine residue. J Cell Sci 2002; 115: 165–173. 11801734 10.1242/jcs.115.1.165

[bib26] Raijmakers R, Kraiczek K, de Jong AP, Mohammed S, Heck AJR. Exploring the human leukocyte phosphoproteome using a microfluidic reversed-phase-TiO2-reversed-phase high-performance liquid chromatography phosphochip coupled to a quadrupole time-of-flight mass spectrometer. Anal Chem 2010; 82: 824–832. 20058876 10.1021/ac901764g

[bib27] Kitada S, Andersen J, Akar S, Zapata JM, Takayama S, Krajewski S et al. Expression of apoptosis-regulating proteins in chronic lymphocytic leukemia: correlations with *In vitro* and *In vivo* chemoresponses. Blood 1998; 91: 3379–3389. 9558396

[bib28] Longo PG, Laurenti L, Gobessi S, Petlickovski A, Pelosi M, Chiusolo P et al. The Akt signaling pathway determines the different proliferative capacity of chronic lymphocytic leukemia B-cells from patients with progressive and stable disease. Leukemia 2007; 21: 110–120. 17024114 10.1038/sj.leu.2404417

[bib29] Longo PG, Laurenti L, Gobessi S, Sica S, Leone G, Efremov DG. The Akt/Mcl-1 pathway plays a prominent role in mediating antiapoptotic signals downstream of the B-cell receptor in chronic lymphocytic leukemia B cells. Blood 2008; 111: 846–855. 17928528 10.1182/blood-2007-05-089037

[bib30] Woyach JA, Johnson AJ, Byrd JC. The B-cell receptor signaling pathway as a therapeutic target in CLL. Blood 2012; 120: 1175–1184. 22715122 10.1182/blood-2012-02-362624 PMC3418714

[bib31] Ghosh AK, Kay NE. Critical signal transduction pathways in CLL. Adv Exp Med Biol 2013; 792: 215–239. 24014299 10.1007/978-1-4614-8051-8_10 PMC3918736

[bib32] Rosati E, Sabatini R, Rampino G, Tabilio A, Di Ianni M, Fettucciari K et al. Constitutively activated Notch signaling is involved in survival and apoptosis resistance of B-CLL cells. Blood 2009; 113: 856–865. 18796623 10.1182/blood-2008-02-139725

[bib33] Saiya-Cork K, Collins R, Parkin B, Ouillette P, Kuizon E, Kujawski L et al. A pathobiological role of the insulin receptor in chronic lymphocytic leukemia. Clin Cancer Res Off J Am Assoc Cancer Res 2011; 17: 2679–2692. 10.1158/1078-0432.CCR-10-2058 PMC3086966 21307146

[bib34] Yaktapour N, Übelhart R, Schüler J, Aumann K, Dierks C, Burger M et al. Insulin-like growth factor-1 receptor (IGF1R) as a novel target in chronic lymphocytic leukemia. Blood 2013; 122: 1621–1633. 23863897 10.1182/blood-2013-02-484386

[bib35] Burger JA, Burger M, Kipps TJ. Chronic lymphocytic leukemia B cells express functional CXCR4 chemokine receptors that mediate spontaneous migration beneath bone marrow stromal cells. Blood 1999; 94: 3658–3667. 10572077

[bib36] Möhle R, Failenschmid C, Bautz F, Kanz L. Overexpression of the chemokine receptor CXCR4 in B cell chronic lymphocytic leukemia is associated with increased functional response to stromal cell-derived factor-1 (SDF-1). Leukemia 1999; 13: 1954–1959. 10602415 10.1038/sj.leu.2401602

[bib37] Ticchioni M, Essafi M, Jeandel PY, Davi F, Cassuto JP, Deckert M et al. Homeostatic chemokines increase survival of B-chronic lymphocytic leukemia cells through inactivation of transcription factor FOXO3a. Oncogene 2007; 26: 7081–7091. 17496928 10.1038/sj.onc.1210519

[bib38] Schröttner P, Leick M, Burger M. The role of chemokines in B cell chronic lymphocytic leukaemia: pathophysiological aspects and clinical impact. Ann Hematol 2010; 89: 437–446. 20020127 10.1007/s00277-009-0876-6

[bib39] Drost AC, Seitz G, Boehmler A, Funk M, Norz KP, Zipfel A et al. The G protein-coupled receptor CysLT1 mediates chemokine-like effects and prolongs survival in chronic lymphocytic leukemia. Leuk Lymphoma 2012; 53: 665–673. 21936770 10.3109/10428194.2011.625578

[bib40] Breitschopf K, Haendeler J, Malchow P, Zeiher AM, Dimmeler S. Posttranslational modification of Bcl-2 facilitates its proteasome-dependent degradation: molecular characterization of the involved signaling pathway. Mol Cell Biol 2000; 20: 1886–1896. 10669763 10.1128/mcb.20.5.1886-1896.2000 PMC85374

[bib41] Rooswinkel RW, van de Kooij B, de Vries E, Paauwe M, Braster R, Verheij M et al. Antiapoptotic potency of Bcl-2 proteins primarily relies on their stability, not binding selectivity. Blood 2014; 123: 2806–2815. 24622325 10.1182/blood-2013-08-519470

[bib42] Tomasi ML, Ryoo M, Ramani K, Tomasi I, Giordano P, Mato JM et al. Methionine adenosyltransferase α2 sumoylation positively regulate Bcl-2 expression in human colon and liver cancer cells. Oncotarget 2015; 6: 37706–37723. 26416353 10.18632/oncotarget.5342 PMC4741959

[bib43] Wu Z, Galmiche A, Liu J, Stadler N, Wendum D, Segal-Bendirdjian E et al. Neurotensin regulation induces overexpression and activation of EGFR in HCC and restores response to erlotinib and sorafenib. Cancer Lett 2017; 388: 73–84. 27914862 10.1016/j.canlet.2016.11.032

[bib44] Magazin M, Poszepczynska-Guigné E, Bagot M, Boumsell L, Pruvost C, Chalon P et al. Sezary syndrome cells unlike normal circulating T lymphocytes fail to migrate following engagement of NT1 receptor. J Invest Dermatol 2004; 122: 111–118. 14962098 10.1046/j.0022-202X.2003.22131.x

[bib45] Choi SY, Chae HD, Park TJ, Ha H, Kim KT. Characterization of high affinity neurotensin receptor NTR1 in HL-60 cells and its down regulation during granulocytic differentiation. Br J Pharmacol 1999; 126: 1050–1056. 10193787 10.1038/sj.bjp.0702378 PMC1571214

[bib46] Swift SL, Burns JE, Maitland NJ. Altered expression of neurotensin receptors is associated with the differentiation state of prostate cancer. Cancer Res 2010; 70: 347–356. 20048080 10.1158/0008-5472.CAN-09-1252

[bib47] Ohman L, Franzén L, Rudolph U, Birnbaumer L, Hörnquist EH. Regression of Peyer’s patches in G alpha i2 deficient mice prior to colitis is associated with reduced expression of Bcl-2 and increased apoptosis. Gut 2002; 51: 392–397. 12171962 10.1136/gut.51.3.392 PMC1773369

[bib48] Dalwadi H, Wei B, Schrage M, Spicher K, Su TT, Birnbaumer L et al. B cell developmental requirement for the G alpha i2 gene. J Immunol 2003; 170: 1707–1715. 12574334 10.4049/jimmunol.170.4.1707

[bib49] Cattaneo F, Guerra G, Parisi M, De Marinis M, Tafuri D, Cinelli M et al. Cell-surface receptors transactivation mediated by g protein-coupled receptors. Int J Mol Sci 2014; 15: 19700–19728. 25356505 10.3390/ijms151119700 PMC4264134

[bib50] El-Shewy HM, Johnson KR, Lee M-H, Jaffa AA, Obeid LM, Luttrell LM. Insulin-like growth factors mediate heterotrimeric G protein-dependent ERK1/2 activation by transactivating sphingosine 1-phosphate receptors. J Biol Chem 2006; 281: 31399–31407. 16926156 10.1074/jbc.M605339200

[bib51] Delcourt N, Bockaert J, Marin P. GPCR-jacking: from a new route in RTK signalling to a new concept in GPCR activation. Trends Pharmacol Sci 2007; 28: 602–607. 18001849 10.1016/j.tips.2007.09.007

[bib52] Mira E, Lacalle RA, González MA, Gómez-Moutón C, Abad JL, Bernad A et al. A role for chemokine receptor transactivation in growth factor signaling. EMBO Rep 2001; 2: 151–156. 11258708 10.1093/embo-reports/kve027 PMC1083823

[bib53] Hobson JP, Rosenfeldt HM, Barak LS, Olivera A, Poulton S, Caron MG et al. Role of the sphingosine-1-phosphate receptor EDG-1 in PDGF-induced cell motility. Science 2001; 291: 1800–1803. 11230698 10.1126/science.1057559

[bib54] Moughal NA, Waters C, Sambi B, Pyne S, Pyne NJ. Nerve growth factor signaling involves interaction between the Trk A receptor and lysophosphatidate receptor 1 systems: nuclear translocation of the lysophosphatidate receptor 1 and Trk A receptors in pheochromocytoma 12 cells. Cell Signal 2004; 16: 127–136. 14607283 10.1016/j.cellsig.2003.08.004

[bib55] Moughal NA, Waters CM, Valentine WJ, Connell M, Richardson JC, Tigyi G et al. Protean agonism of the lysophosphatidic acid receptor-1 with Ki16425 reduces nerve growth factor-induced neurite outgrowth in pheochromocytoma 12 cells. J Neurochem 2006; 98: 1920–1929. 16945108 10.1111/j.1471-4159.2006.04009.x

[bib56] Vita N, Oury-Donat F, Chalon P, Guillemot M, Kaghad M, Bachy A et al. Neurotensin is an antagonist of the human neurotensin NT2 receptor expressed in Chinese hamster ovary cells. Eur J Pharmacol 1998; 360: 265–272. 9851594 10.1016/s0014-2999(98)00678-5

[bib57] Bürkle A, Niedermeier M, Schmitt-Gräff A, Wierda WG, Keating MJ, Burger JA. Overexpression of the CXCR5 chemokine receptor, and its ligand, CXCL13 in B-cell chronic lymphocytic leukemia. Blood 2007; 110: 3316–3325. 17652619 10.1182/blood-2007-05-089409

[bib58] De la Cruz-Morcillo MA, Berger J, Sánchez-Prieto R, Saada S, Naves T, Guillaudeau A et al. p75 neurotrophin receptor and pro-BDNF promote cell survival and migration in clear cell renal cell carcinoma. Oncotarget 2016; 7: 34480–34497. 27120782 10.18632/oncotarget.8911 PMC5085170

[bib59] Sun C-Y, Chu Z-B, She X-M, Zhang L, Chen L, Ai L-S et al. Brain-derived neurotrophic factor is a potential osteoclast stimulating factor in multiple myeloma. Int J Cancer 2012; 130: 827–836. 21400510 10.1002/ijc.26059

[bib60] Cazorla M, Prémont J, Mann A, Girard N, Kellendonk C, Rognan D. Identification of a low-molecular weight TrkB antagonist with anxiolytic and antidepressant activity in mice. J Clin Invest 2011; 121: 1846–1857. 21505263 10.1172/JCI43992 PMC3083767

[bib61] Strober W. Trypan Blue Exclusion Test of Cell Viability. Curr Protoc Immunol 2015; 111: A3.B.1–3. 26529666 10.1002/0471142735.ima03bs111 PMC6716531

[bib62] Bellanger C, Dubanet L, Lise M-C, Fauchais A-L, Bordessoule D, Jauberteau M-O et al. Endogenous neurotrophins and Trk signaling in diffuse large B cell lymphoma cell lines are involved in sensitivity to rituximab-induced apoptosis. PloS One 2011; 6: e27213. 22076137 10.1371/journal.pone.0027213 PMC3208602

