# Supplementary material for: Neurotensin receptor type 2 protects B-cell chronic lymphocytic leukemia cells from apoptosis
Source: Oncogene. 2017 Oct 23;37(6):756–67. doi: 10.1038/onc.2017.365 (PMC5808079; doi:10.1038/onc.2017.365)
Supplement: Supplementary Figure 1 [file onc2017365x1.pdf]

Supplementary Figure 1

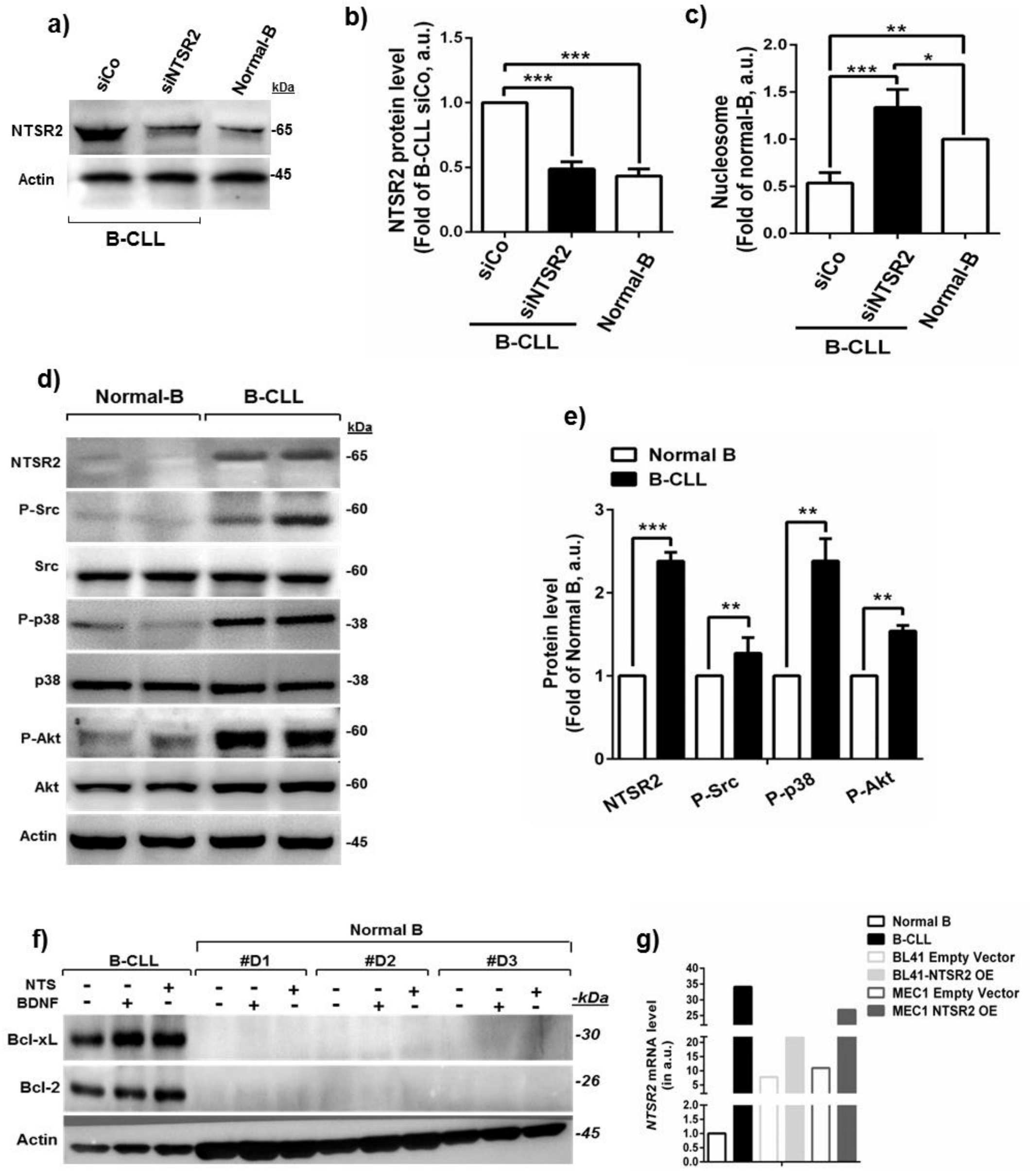

Supplementary Figure 1. Survival signaling pathways in B-CLL and normal B cells
(a) Western blot of NTSR2 expression from B-CLL cell lysates, depleted of *NTSR2* or not for 72 h *vs*. Normal B cells. **(b)** histograms: fold change in NTSR2 expression in si*NTSR2* cells, siControl (siCo) and in Normal B, normalized against actin **(c)** Apoptotic ratio (nucleosome) in B-CLL cells (n=3) depleted of *NTSR2* or not for 72 h *vs*. Normal B (n=3), expressed as fold change vs. control (siCo). **(d, e)** Western blot analysis of NTSR2, Src, p38MAPK, and Akt expression from B-CLL lymphocytes vs. normal B lymphocytes, expressed as mean fold change in expression (± s.e.m.) in comparison with normal B cells. Values: means ± s.e.m. of three independent experiments, in arbitrary units (a.u.). **(f)** western blot of Bcl-xL and Bcl-2, comparing B lymphocytes from three normal donors (#D1, #D2, #D3) and one B-CLL patient (#P1). **(g)** *NTSR2* mRNA level in B-CLL lymphocytes (n=30) vs. normal B lymphocytes (n=15), and in BL-41 or MEC-1 cells transfected with NTSR2 expression vector (*p*CMV6 NTSR2) (n=15) vs. empty vector (EV) (n=15). Data are expressed as mean fold change in expression (± s.e.m.), with the value from normal B cells defined as 1**.**
