# Supplementary material for: Neurotensin receptor type 2 protects B-cell chronic lymphocytic leukemia cells from apoptosis
Source: Oncogene. 2017 Oct 23;37(6):756–67. doi: 10.1038/onc.2017.365 (PMC5808079; doi:10.1038/onc.2017.365)
Supplement: Supplementary Figure 2 [file onc2017365x2.pdf]

Supplementary Figure 2

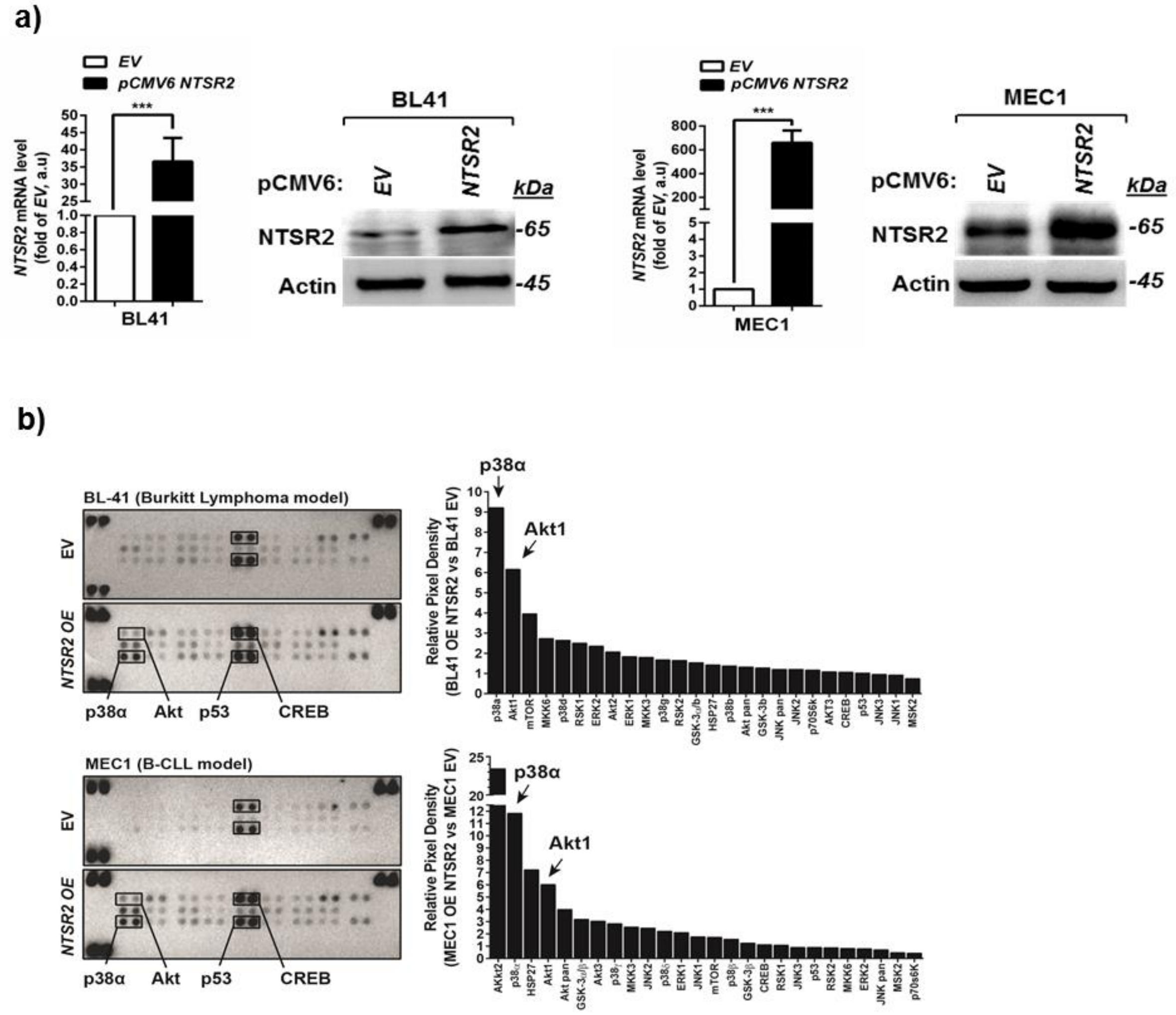

Supplementary Figure 2. NTSR2 overexpression in BL-41 and MEC-1 cells

(a) *NTSR2* mRNA and NTSR2 protein expression levels in BL-41 or MEC-1 cells transfected with NTSR2 expression vector (pCMV6 NTSR2) or empty vector (EV). Data are expressed as mean fold change (± s.e.m.) vs. empty vector. All experiments were repeated at least three times. **(b)** The signaling pathways activated by *NTSR2* overexpression were investigated using the Proteome Profiler Human Phospho-MAPK Array Kit (R&D Systems). In brief, transfected cells were grown in 24-well plates for 24 h and lysed, and approximately 300 µg total protein was analyzed in each assay. Values are expressed as mean fold changes in NTSR2 expression with respect to cells transfected with the empty vector.
