# Supplementary material for: Neurotensin receptor type 2 protects B-cell chronic lymphocytic leukemia cells from apoptosis
Source: Oncogene. 2017 Oct 23;37(6):756–67. doi: 10.1038/onc.2017.365 (PMC5808079; doi:10.1038/onc.2017.365)
Supplement: Supplementary Figure 3 [file onc2017365x3.pdf]

Supplementary Figure 3

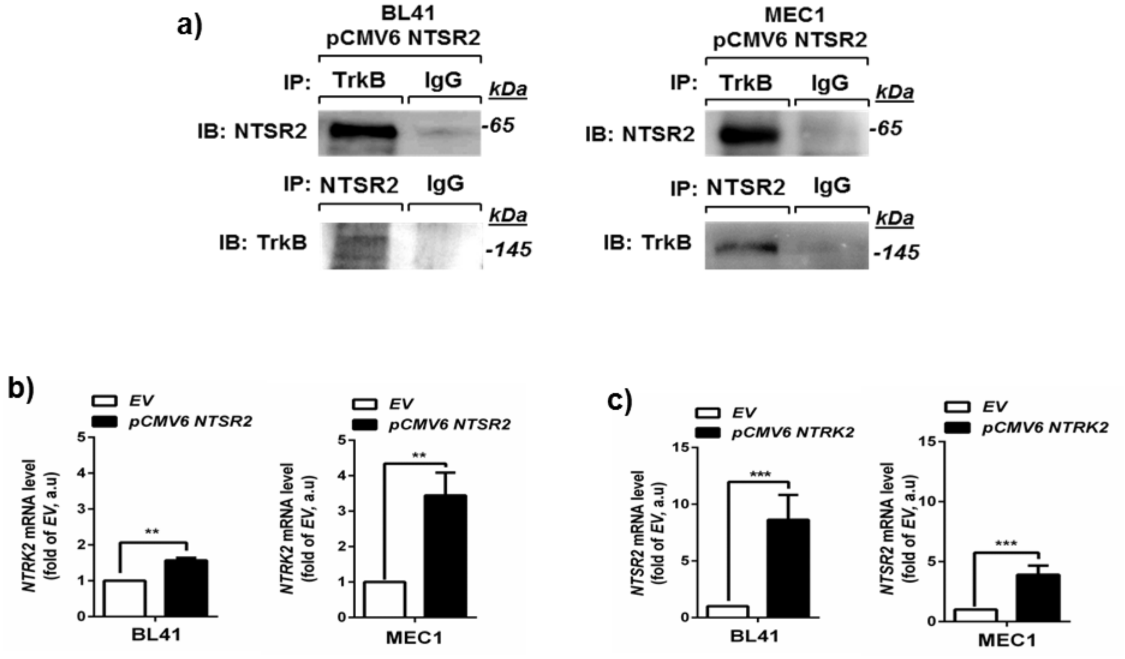

Supplementary Figure 3. NTSR2 and TrkB Interactions in BL-41 and MEC-1 cells
(a) Immunoprecipitation (IP) of TrkB or NTSR2 from BL-41 and MEC-1 cells overexpressing NTSR2 followed by immunoblot (IB) with indicated antibodies. All experiments were repeated at least three times; representative results are shown. **(b)** Quantitative analyses of *NTRK2* mRNA level in BL-41 or MEC-1 cells transfected with NTSR2 expression vector (pCMV6 NTSR2) or empty vector (EV). **(c)** Quantitative analyses of *NTSR2* mRNA level in BL-41 or MEC-1 cells transfected with NTRK2 expression vector (pCMV6 NTRK2) or empty vector (EV). Values are mean change in *NTSR2* or *NTRK2* expression (± s.e.m.) vs. empty vector. All experiments were repeated at least three times.
