# Supplementary material for: Neurotensin receptor type 2 protects B-cell chronic lymphocytic leukemia cells from apoptosis
Source: Oncogene. 2017 Oct 23;37(6):756–67. doi: 10.1038/onc.2017.365 (PMC5808079; doi:10.1038/onc.2017.365)
Supplement: Supplementary Figure 4 [file onc2017365x4.pdf]

Supplementary Figure 4

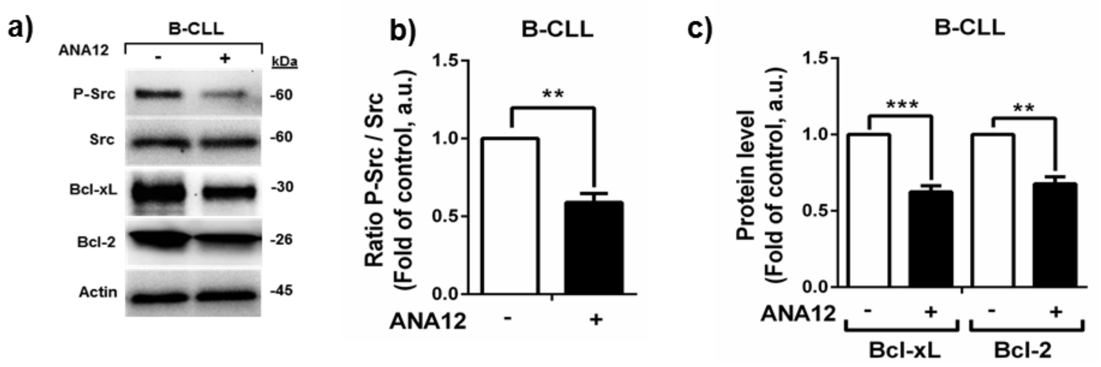

Supplementary Figure 4. TrkB inhibition and prosurvival pathways

(a) Western blot analysis of P-Src, Bcl-xL, and Bcl-2 in B-CLL cell lysates following TrkB inhibition with ANA12 at 100 µM for 24 h. **(b, c)** Expression levels of p-Src **(b),** Bcl-xL, and Bcl-2 **(c)** represented as, respectively, the ratio of phosphorylated Src vs. pan-Src protein and the ratios of Bcl-xL and Bcl-2 to actin (B-CLL, n=3). Values are means ± s.e.m. of three independent experiments, in arbitrary units (a.u.).
