# Supplementary material for: Neurotensin receptor type 2 protects B-cell chronic lymphocytic leukemia cells from apoptosis
Source: Oncogene. 2017 Oct 23;37(6):756–67. doi: 10.1038/onc.2017.365 (PMC5808079; doi:10.1038/onc.2017.365)
Supplement: Supplementary Figure 5 [file onc2017365x5.pdf]

Supplementary Figure 5

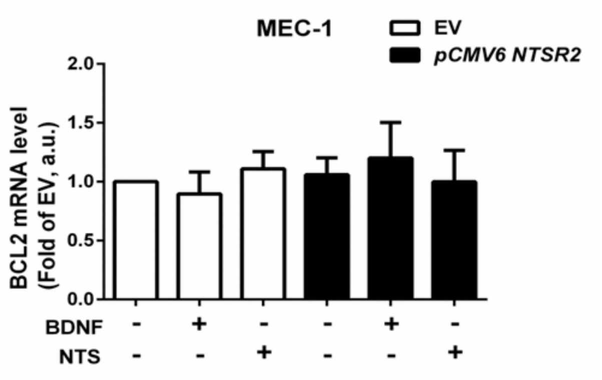

Supplementary Figure 5. BDNF or NTS stimulation did not modified *BCL2* mRNA levels in NTSR2-overexpressing MEC-1 cells

Quantitative analyses of *BCL2* mRNA levels in MEC-1 cells transfected with NTSR2 expression vector (pCMV6 NTSR2) or empty vector (EV) after addition of BDNF (100 ng/mL) and neurotensin (40 µM) for 24 h. Data are expressed as mean fold change in expression (± s.e.m.) in comparison with EV transfected cells without treatments. Values are means ± s.e.m. of three independent experiments, in arbitrary units (a.u.).
